# Sex Education in Italy: An Overview of 15 Years of Projects in Primary and Secondary Schools

**DOI:** 10.1007/s10508-023-02541-6

**Published:** 2023-02-07

**Authors:** Giuseppina Lo Moro, Fabrizio Bert, Toni Cappelletti, Heba Safwat Mhmoued Abdo Elhadidy, Giacomo Scaioli, Roberta Siliquini

**Affiliations:** 1 Department of Public Health Sciences, University of Turin, Via Santena 5 Bis, 10126 Turin, Italy; 2 A.O.U. City of Health and Science of Turin, Turin, Italy

**Keywords:** Sex education, Schools, Children, Adolescents, Health promotion

## Abstract

School-based sex education is essential to improve the knowledge and behavior of young people. Unlike most European countries, sex education in Italy is not compulsory in the school curriculum. Therefore, to assess potential gaps, the main aim of this study was to explore Italian regional initiatives about sex education. A review of gray literature from 2006 to 2021 was conducted in December 2021 on national databases and institutional websites of each Italian region, during which official documents on regional sex education programs were selected. Moreover, a rapid systematic review was carried out in scientific databases to find more information. For both reviews, the sex education topics recommended by UNESCO were used for extracting and analyzing data. A total of 12 Italian regions out of 20 had at least one sex education program, for a total of 39 projects. Overall, all the topics identified by UNESCO have been covered, with considerable differences between northern, central, and southern Italy. Contraception, love, marriage, partnerships, and family were the main topics discussed (hosted in 92% of the regions). Only 25% of the regions discussed pregnancy and birth. Disability was the least debated topic (17%). The rapid systematic review showed that relevant improvements were achieved after sex education interventions. In conclusion, this study underlined important limitations to the implementation of school-based sex education in Italy. Hence, it is necessary to increase sex education interventions to reach adequate standards and flatten the differences not only between Italian regions but also between Italy and other European countries.

**Supplementary Information:**

The online version contains supplementary material available at 10.1007/s10508-023-02541-6.

## Introduction

The World Health Organization (WHO) defines sexuality as a central aspect of human beings, which includes many factors such as sex, gender, sexual orientation, eroticism, and reproduction. There is an important interaction between sexuality and biological, psychological, social, economic, political, and religious factors (WHO, n.d.). Given its role in people’s lives, sexuality should be taught to children, teenagers, and young adults so that they can have a healthy sexual life (UNESCO, 2018). Over the last few decades, various studies have shown that teaching young people about sexuality can have positive impacts on them, especially in regard to delaying first intercourse and increasing the use of condoms or other contraceptive methods, which reduces sexually transmitted diseases (STDs) and unwanted pregnancies (De Santi, Guerra, & Morosini, 2008; García-Vázquez et al., 2020; Vivancos et al., 2013). Other outcomes of sex education include violence prevention, the development of healthy relationships, improved social and emotional learning, and increased media literacy (Goldfarb & Lieberman, 2020). The evidence also strongly suggests that sex education does not have negative effects on youth; specifically, sex education does not increase harmful sexual behaviors, despite public opinion beliefs (Kirby et al., 2007).

In this respect, in 2009, the United Nations Educational, Scientific and Cultural Organization (UNESCO) published the “International Technical Guidance on Sexuality Education” guidelines, with the intention of providing concrete guidance for the development of locally adapted curricula (UNESCO, 2009). These guidelines, as well as the available scientific evidence on sex education, suggest that the school environment is an ideal setting in which to promote health (De Santi et al., 2008; Future of Sex Education Initiative, 2012; Vivancos et al., 2013). Substantial evidence supports the establishment of sex education programs in primary schools and long-term programs (Goldfarb & Lieberman, 2020). In particular, the approach taken to teaching sex education should include the intervention of professionals trained in different areas so that there is available comprehensive information that covers the physiological and relational aspects of sexuality (European Parliament, 2013).

The member states of the European Union (EU) do not have a common policy about sex education. In most EU member states, sex education is mandatory, except for Bulgaria, Cyprus, Italy, Lithuania, Poland, and Romania (Stull, 2012). With regard to Italy, a report by the International Planned Parenthood Federation (IPPF) European Network published in 2006 showed that sex education has the opposition of the Catholic Church and of some political groups, despite numerous proposals that have taken place over the last 30 years; furthermore, both public opinion and official attitudes tend to be traditional and conservative (IPPF, 2006). Indeed, the 1984 Concordat between the Catholic Church and the Italian government stipulated that the Ministry of Education must consider the views of the Church (European Parliament, 2013). In 1991, a bill designed to ensure that sex education would be incorporated into biology lessons did not become an effective law. Since 1995, a series of legislative proposals to introduce sex education into school programs have been unsuccessful (Fontana, 2018). In addition, beyond the potential differences between Italy and other EU members, it is important to consider that there might be many disparities between the Italian regions. Indeed, Italy can be subdivided into three geographic areas (north, center, and south) in consideration of their economic, cultural, and social backgrounds (OECD, 2020). In particular, a recent European report on inequalities in Italy confirmed the presence of territorial differences spreading throughout the country, placing the south at a disadvantage on multiple levels (Pastorelli & Stocchiero, 2019).

In this context, several studies conducted among Italian adolescents aiming to assess their knowledge about sexuality have shown that there is an urgent need for sex education (Bergamini et al., 2013; Bogani et al., 2015; Borraccino et al., 2020; Drago et al., 2016; Orlando et al., 2019; Smorti et al., 2019). In particular, Drago et al. showed that the majority of enrolled students believe that the school should play a central role in this educational process. However, despite this awareness, most of these students rated the sex education received at their school as either poor or absent. Furthermore, looking at the issue from another perspective, data from the National Surveillance on STDs, which has been active since 1991, show that in Italy, the STD incidence rate remained stable until 2004, while from 2005 to 2019, it increased by 41.8% (Epicentro, n.d.).

These data highlight a substantial dysfunction in the Italian educational system. Given the importance of sex education and considering the lack of clear legislation on the subject (European Parliament, 2013), it is worth exploring the issue. Despite the general picture outlined, in Italy, local authorities can plan and implement sex education programs. However, to our knowledge, there is no current study or report aimed at evaluating and summarizing these programs. Moreover, given the Italian scenario of sex education, the effectiveness of the programs implemented in this specific context might be questioned, despite the demonstrated effectiveness of programs that follow international guidelines (De Santi et al., 2008; García-Vázquez et al., 2020; Goldfarb & Lieberman, 2020; Vivancos et al., 2013).

Therefore, the primary aim of this study is to evaluate the amount and characteristics of Italian local initiatives about school-based sex education over a span of 15 years. The main research questions that the present paper addresses are as follows: (1) Are these local initiatives in alignment with the topics recommended by UNESCO standards, or is Italy truly lacking in this field? (2) Are there regional differences within Italy in regard to the sex education programs offered? (3) Is the effectiveness of sex education interventions assessed, and, if so, are these interventions effective?

## Method

To retrieve documents about Italian sex education programs, a double strategy composed of a review of gray literature (based on programs included in national databases) and a rapid systematic review on scientific databases was carried out. The evaluation was limited to the period ranging from 2006 to 2021 due to the relevant changes that have occurred in the field of sex education since those years, such as the IPPF report (IPPF, 2006) and UNESCO’s technical guidelines (UNESCO, 2009), which were published in 2006 and 2009, respectively, and the substantial changes that have occurred in the incidence of STDs in Italy (Epicentro, n.d.).

### Review of Gray Literature

#### Data Sources and Search Strategy

The authors searched for sex education programs based on regional partnerships between schools and local public health units. All Italian regions, subdivided into northern, central, and southern regions by using the Nomenclature of Territorial Units for Statistics (NUTS) (European Commission, 2011), were evaluated to provide an overview of school-based sex education. The search was conducted on PRO.SA (a national database of projects, interventions, policies, and good practices of health prevention and promotion that aims to document, share, and network projects and their results to support the activities of operators, decision-makers, and stakeholders) and on public health unit/region websites of each Italian region, focusing on official documents containing training catalogs for schools that used the Italian terms for "sex education."

The search strategy was structured on the population, intervention, comparison, and outcome (PICO) model, as presented in Table 1. The search was performed in December 2021 and included documents produced between 2006 and 2021.

**Table 1:** PICO model used for the search of relevant studies

Population	Students attending primary and secondary schools in Italy
Intervention	Sex education projects
Comparison	Any
Outcome	Any

#### Document Selection and Data Extraction

Documents were considered eligible if they reported sex education programs based on regional partnerships with public health units and offered in schools. The documents were excluded if (1) the program was performed/planned in the 2021–2022 academic year, as this academic year was still in progress when this study was conducted; (2) the program was promoted by private and/or nongovernmental associations to gather only institutional data; (3) the program was dedicated to university students, as they are out of the compulsory school system; (4) the program was characterized by meetings aimed only at promoting health care services relating to sexual health; and (5) the program did not mention data about either the promoter or the target.

Only documents written in Italian were deemed eligible. Documents were included if the targets of the program were students and/or parents and/or teachers (in a primary/secondary school setting). Two authors selected the documents and extracted the data. Any disagreement was resolved by discussion and consensus with a third author. For each project, the extracted data included the (1) year/s of implementation; (2) promoting institution; (3) location; (4) target; (5) involved professional figures; (6) total hours; (7) covered topics; and (8) presence and results of a final evaluation.

The main topics of sex education that were evaluated were those described by UNESCO (Goldfarb & Lieberman, 2020): (1) biological aspects, body image, puberty, and anatomy; (2) love, marriage, relationships, and family; (3) sexual/domestic abuse and gender-based violence; (4) pregnancy and birth; (5) sexual orientation and lesbian, gay, bisexual, transgender, and other sexual and gender minorities (LGBT +) issues; (6) HIV/AIDS and STDs; (7) contraception; (8) gender roles; (9) mutual consent; (10) human rights; and (11) online media. Considering recent literature (Holland-Hall & Quint, 2017), the topic “disability and sexuality” was also included during the data extraction. The evaluation consisted of the assessment of the presence or absence of each topic in the program.

After the data extraction, a map chart describing the number of projects found per region was created using Microsoft Excel. Due to the heterogeneity of the documents and the characteristics of the extracted data, which were not suitable for combining quantitatively, the authors carried out a narrative synthesis, which is an approach that relies mainly on the use of text to summarize the results (Popay et al., 2006); the extracted data are then presented in Summary of Findings tables. The methodology of narrative synthesis was informed by the work of Popay et al. (2006), and it consisted primarily of two elements. The first element was developing a preliminary synthesis through the use of tools such as textual descriptions and tabulations. Then, the second element was exploring relationships in data, using Microsoft Excel to create pivot cross-tabulations where the topics, the year/s of implementation, the promoting institution, the location, and the target were cross-referenced.

### Rapid Systematic Review in Scientific Databases

#### Data Sources and Search Strategy

To find further information, a rapid systematic review (Khangura et al., 2012) of the literature was performed by searching the Scopus and PubMed databases and reported following the Preferred Reporting Items for Systematic Reviews and Meta-Analyses (PRISMA) checklist (Page et al., 2021). The search strings were as follows:

1. Scopus: TITLE-ABS-KEY (( "sex education" OR ( sexual* AND education)) AND Ital*);
2. PubMed: ("Sex Education"[Mesh] OR "sex education"[tiab] OR (sexual*[tiab] AND education [tiab])) AND Ital*[tiab]).

In both cases, a time limit covering the years from 2006 to 2021 was set. The search was performed on December 21, 2021. The PICO model used was the same as that used in the review of gray literature.

#### Document Selection and Data Extraction

The inclusion and exclusion criteria were the same as those used in the review of gray literature, except for the language; in this case, documents written in English and Italian were included.

The authors chose the Qatar Computing Research Institute (QCRI) web application Rayyan (Ouzzani et al., 2016) as the tool for selecting relevant studies. Two authors independently screened the titles and abstracts to identify appropriate studies and applied the inclusion and exclusion criteria to full texts. Divergences were resolved by consensus of three authors, and the reasons for full-text exclusion were documented. In addition to the data extracted from local projects, the following categories were identified: (1) year of publication; (2) journal; (3) sample size; (4) presence and size of the control group; and (5) type of intervention.

## Results

### Review of Gray Literature

Considering all 20 Italian regions, eight (Abruzzo, Apulia, Calabria, Liguria, Marche, Sardinia, Sicily, and Tuscany) were excluded because no projects met the inclusion criteria. The remaining 12 regions (Aosta Valley, Basilicata, Campania, Emilia-Romagna, Friuli-Venezia Giulia, Lazio, Lombardy, Molise, Piedmont, Trentino-South Tyrol, Umbria, and Veneto) each had at least one project that met the inclusion criteria.

**Fig. 1:**
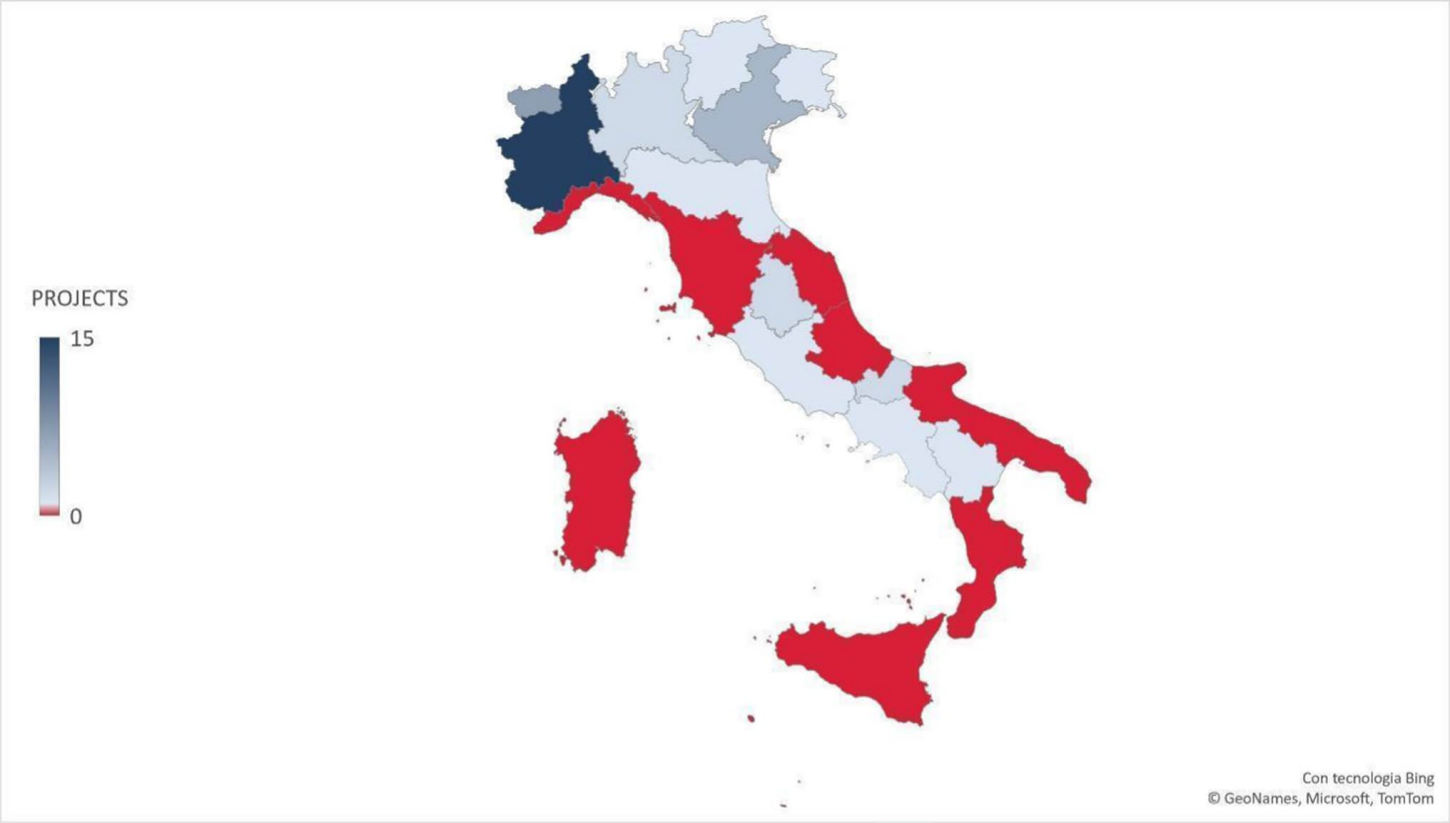
Number of projects about sex education in primary and secondary schools for each Italian region implemented from 2006 to 2021 (Microsoft Excel file)

The total number of projects was 39: 32 in northern Italy, three in central Italy, and four in southern Italy. In Fig. 1, the regions without projects are colored in red, while a different gradation of blue reflects the number of projects in the other regions, ranging from one to 15.

Most of the projects (*n* = 23) were carried out only once; eight were created and completed in the single year of 2012, five were created and completed in 2017, three were created and completed in 2006, and three were created and completed in 2008. Other projects were carried out in 2007 (*n* = 1), 2013 (*n* = 1), 2016 (*n* = 1), and 2019 (*n* = 1). The remaining 16 projects were repeated for at least two years in a row; the longest running project started in 2006 and has been consistently repeated every year. All the projects conducted in the center of Italy were performed for a single year, while the projects conducted in the south lasted two or three years. In the north, the duration of the projects was variable, ranging from one year to 15 years.

Considering the promoting institution, 29 projects were realized by the ASL (Azienda Sanitaria Locale), which is a local health unit, and 10 were realized by regions. Three projects included visits to a counseling center, while the rest were hosted only by schools.

The target of the projects was represented mostly by students. A total of 34 projects included lessons for either students only or for students and parents/teachers. Three projects were addressed only to teachers, one only to parents, and one to both parents and teachers to provide them with knowledge about how to educate children or adolescents.

The professional figures involved in programs were represented as follows: 23 projects were conducted by health professionals, one was conducted by medical students, one was conducted by Italian Red Cross professionals, 12 were conducted by psychologists, four were conducted by teachers, five were conducted by social assistants, and one was conducted by sociologists (several programs were conducted by more than one professional figure). This information was not available for retrieval for 11 projects. The number of hours for every project was variable; the shortest lasted two hours, and the longest lasted 182 h. Further information about the characteristics of the projects can be found in Appendix Table A1 (Appendix A), while the references of all projects are provided in Appendix Table B1 (Appendix B).

The distribution of the topics in each project is reported in Appendix Table A2 (Appendix A). Table 2 shows the topics covered in each region, summing up the projects conducted in each specific region. We found that all of the UNESCO topics were covered but to varying degrees in the different regions, with notable differences between northern, central, and southern Italy.

**Table 2:** Topics about sexuality covered in sex education programs implemented in primary and secondary schools in each Italian region from 2006 to 2021

Position	Region	Biological aspects/body awareness/puberty and anatomy	Love, marriage, partnerships, family	Sexual/domestic abuse and gender-based violence	Pregnancy and birth	Sexual orientation/lgbti issues	Hiv/Aids and stis	Contraception	Gender roles	Mutual consent	Human rights	Online media	Disability
North	Emilia-Romagna	X	X				X	X	X				
North	Friuli-Venezia Giulia	X	X				X	X					
North	Lombardy		X				X	X					
North	Piedmont	X	X	X	X	X	X	X	X				
North	Trentino-south tyrol	X	X				X	X			X		
North	Aosta valley	X	X	X	X	X	X	X	X	X	X	X	X
North	Veneto	X	X	X		X		X	X			X	X
Center	Lazio						X						
Center	Umbria	X	X			X		X	X	X			
South	Basilicata	X	X	X	X		X	X		X	X		
South	Campania	X	X	X		X	X	X	X	X	X	X	
South	Molise	X	X				X	X				X	

Contraception, along with love, marriage, partnerships, and family, were the main topics discussed during sex education courses in schools (hosted in 92% of the regions), followed by biological aspects, body awareness, puberty, and anatomy and HIV/AIDS and STDs (83%). In approximately half of the regions, gender roles (50%), sexual orientation or LGBT + issues and sexual/domestic abuse and gender-based violence (42%) were discussed in classes. Lectures about mutual consent, human rights, and online media were less common; only 33% of the regions covered these topics. Only 25% of the regions discussed pregnancy and birth. The least debated topic was disability; only 17% of the regions talked about sexuality in regard to disabled people.

In 64% of cases, there was a final evaluation used to assess the learning of students; however, it was impossible to find material online about the results of these tests.

In northern Italy, all the included regions talked about love, marriage, partnerships, family, and contraception, while 86% included information about biological aspects, body awareness, puberty, anatomy, and HIV/AIDS and STDs. A total of 57% of the northern regions discussed contraception and gender roles, and 43% discussed sexual/domestic abuse, gender-based violence and sexual orientation/LGBT + issues. In addition, 29% of these regions dealt with pregnancy and birth, human rights, online media, and disability. The least discussed topic was mutual consent, which was presented in only 14% of the northern regions.

Considering the included central regions, 50% discussed biological aspects, body awareness, puberty and anatomy, love, marriage, partnerships, family, sexual orientation/LGBTI issues, contraception, HIV/AIDS and STDs, and mutual consent. Sexual/domestic abuse and gender-based violence, pregnancy and birth, human rights, online media, and disability were not discussed.

In the south, the topics discussed in all the included regions were biological aspects, body awareness, puberty and anatomy, love, marriage, partnerships, family, contraception, and gender roles. In 67% of these regions, topics such as sexual/domestic abuse and gender-based violence, mutual consent, human rights, and online media were touched upon. Few regions (33%) discussed pregnancy and birth, sexual orientation/LGBT+ issues, HIV/AIDS, and STDs. No regions mentioned the topic of disability.

The cross-tabulations showed that mutual consent was treated from 2006 to 2012 and that no projects included the topic until 2018; a similar pathway was found for human rights, starting in 2007. The topic of online media was mentioned in 2012 but not again until 2017. Disability was treated occasionally, i.e., in 2006, 2012 and 2017. The projects promoted by regions were targeted to students at secondary schools, while the ones promoted by the ASL also included primary school students. In addition, unlike the projects carried out in schools, those that included visits to a counseling center were suitable only for students at secondary schools. Regarding the targets, the topics of disability, human rights, and mutual consent were expounded only for secondary school students. No other relevant differences were found (data not shown in tables).

### Rapid Systematic Review

**Fig. 2:**
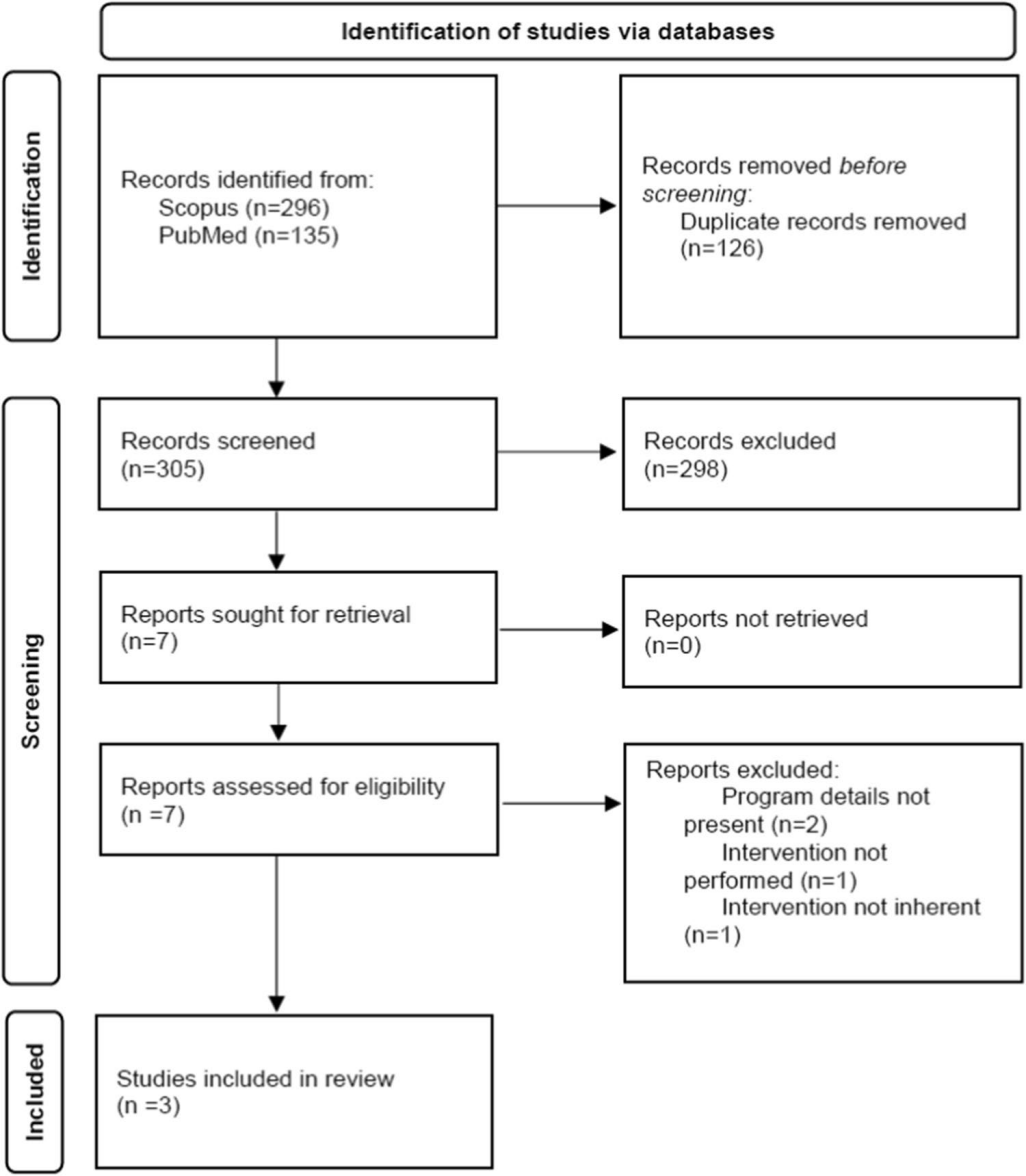
PRISMA 2020 flow diagram for systematic reviews

A total of 296 studies were found on Scopus, and 135 were found on PubMed. Seven studies were selected for full-text screening, but only three of them, detailed in Appendix Table A3 (Appendix A), were finally considered eligible. Data about identification, screening, and inclusion are shown in Fig. 2 according to the PRISMA flow diagram (Page et al., 2021).

The three studies were published in 2012 (Del Prete et al., 2012), 2016 (Benni et al., 2016), and 2021 (Zizza et al., 2021). Two studies were set in southern regions (Del Prete et al., 2012; Zizza et al., 2021), and one was set in a northern region (Benni et al., 2016). All the interventions were carried out only once, on an annual basis, and the setting was the school. The targets were students at lower secondary schools for one project (Del Prete et al., 2012) and at upper secondary schools for the other two projects (Benni et al., 2016; Zizza et al., 2021). The programs were not addressed to parents and teachers. In all the studies, the professional figures involved were external to school staff, i.e., health professionals (Del Prete et al., 2012), medical students, postgraduate students, academic researchers (Benni et al., 2016), and experts (Zizza et al., 2021). The total number of hours was not known for one project (Zizza et al., 2021), while for the other two, it was four (Benni et al., 2016) and five (Del Prete et al., 2012) hours.

HIV/AIDS and STDs were the only topics covered by all three programs. Two out of three programs included lessons about biological aspects, body image, puberty, and anatomy or about contraception (Benni et al., 2016; Del Prete et al., 2012). In one case, the following aspects were discussed: love, marriage, relationships, family, sexual/domestic abuse, gender-based violence, mutual consent, and online media (Del Prete et al., 2012). None of the other topics (pregnancy and birth, sexual orientation, LGBT + issues, gender roles, human rights, disability) were discussed during the interventions (Appendix Table A4, Appendix A).

In each study, surveys were performed before and after the intervention to assess whether there was an improvement in knowledge among students. Two out of the three studies had an experimental and a control group (Benni et al., 2016; Del Prete et al., 2012). Significant changes from the preintervention to postintervention test were revealed. Specifically, in the study by Del Prete et al. (2012), an ad hoc 27-item questionnaire showed that after the course, the students were more likely to use condoms (*p* = 0.002), were aware that STDs can be contracted even after incomplete sexual intercourse (*p* < 0.001) and were more likely to turn to their own parents when they had doubts about sexual issues (*p* = 0.004). In the study by Benni et al., improvements were evaluated with a 16-item questionnaire built using validated questions from other surveys. The analysis underlined significant changes (*p* < 0.001), with strong improvements for all knowledge items in the group that followed the course (Benni et al., 2016). In the study by Zizza et al. (2021), the evaluation consisted of a 35-item ad hoc questionnaire; an improvement in knowledge was found, with significant differences in 60% of the items. More than 94% of the students considered it useful to receive information about sex. The course generated awareness and safety in more than 85% of the students, who perceived a great risk of being infected with HIV/STDs, although pregnancy was seen as a more hazardous consequence of unprotected sex.

## Discussion

The primary aim of the present study was to assess the number and features of Italian local initiatives of sex education in schools over the last 15 years. This paper explored whether the local initiatives were consistent with the topics recommended by UNESCO standards, whether there were differences within the geographical areas of Italy, and whether the implemented programs had been evaluated and, if so, if they were found to be effective in the Italian context.

Taking into consideration the main topics covered during the courses, according to the categories suggested by UNESCO, it should be noted that the most discussed subjects were contraception, love, marriage, partnerships, and family, followed by biological aspects, body awareness, puberty, anatomy, and HIV/AIDS and STDs. In contrast, the less discussed topics were pregnancy and birth, mutual consent, human rights, online media, and disability. A comparison with other European countries showed that in countries such as Austria and the Czech Republic, sex education has a central role, and all the topics concerning sexuality are covered. In contrast, countries such as Estonia and Hungary do not encourage discussion about several sexual topics in schools (European Commission, 2021). In addition, our study showed that some topics, such as disability, human rights, and mutual consent, were exclusively addressed to students at secondary schools. The differentiation of topics according to age levels has also been reported in other countries, suggesting that it is common to have different targets for different topics. For instance, in England, it is mandatory to teach anatomy, puberty and the biological aspects of sexual reproduction to primary and secondary school pupils and to teach about STDs and HIV/AIDS in secondary schools (Pound et al., 2016).

Moreover, most of the projects were carried out in schools; as suggested by the literature, this setting is the key to the implementation of effective sex education (Lameiras-Fernández et al., 2021). Considering that sexual debut usually occurs during adolescence and is increasingly taking place at earlier ages, adolescents need to increase their knowledge about sexuality; however, the literature indicates that they do not receive enough information from parents or other formal sources (Blanc Molina & Rojas Tejada, 2018; Helmer et al., 2015; Lindberg et al., 2016; Magnusson et al., 2019). In this study, the classes were hosted by professional figures linked to physical and mental health; this result is in line with the literature, which asserts that sex education administered during adolescence should be delivered by teachers, parents, health professionals, or community educators (Garcia & Fields, 2017; UNESCO, 2018). As outlined in the results, the number of hours for every project was variable; the cross-tabulations showed that this was not related to the involved professionals or the covered topics. A correlation with the funding provided by the ASL or the regions could be hypothesized, but it was not possible to explore this theory due to the lack of data.

With regard to differences within Italy, the first relevant finding concerns the lack of projects in eight regions, mainly in central and southern Italy. Considering the remaining 12 regions, major discrepancies in the number of training proposals and the covered topics were observed between the north, center, and south regions. The literature is in line with this result, showing relevant differences between northern and southern Italy in several fields, mainly due to substantial cultural differences in the country (Drago et al., 2016; OECD, 2020; Pastorelli & Stocchiero, 2019; Scaioli et al., 2015).

In addition, the projects carried out by each region were uneven both quantitatively and temporally; regarding the number of projects, some regions developed several projects, while others promoted only one program within the same timeframe. Moreover, in some regions there has been a continuity of projects over the years, while other regions have developed individual projects that have not been repeated over the years. The reasons for these disparities are not clear, but they may be due to the success or otherwise of the individual projects. It was not possible to assess data about the effectiveness of the projects since the results of the final evaluations were not available.

Last, considering the rapid systematic review, relevant results have been achieved regarding the knowledge of students before and after the classes about sex education, as students were able to recognize STDs and correctly approach contraceptive methods. Moreover, students considered the courses useful and were more likely to talk about sex and expose their doubts to their parents (Benni et al., 2016; Del Prete et al., 2012; Zizza et al., 2021). Therefore, these findings suggest not only that these programs could be effective but also that Italian students could be receptive and interested in sex education courses. However, it should be noted that the evaluation of the included programs comprised only the assessment of knowledge about sex education. Theories about health promotion suggest that an effective health promotion program should increase not only knowledge but also personal skills and competences (WHO, 2021, n.d.). This is because not all people change their behaviors after simply acquiring new knowledge about a topic. Therefore, this increased level of knowledge among Italian students about sex and sexuality, as highlighted in the abovementioned studies, could not solely reflect a real decrease in sex-related harmful behavior and an increase in sex-related quality of life.

### Limitations

This research had several limitations. First, the search for projects was carried out exclusively online. Although only projects from official sources were selected, it is possible that not all the programs were placed online. Second, there was a lack of information on the effectiveness of the projects due to the absence of the results of the final evaluation. Moreover, in consideration of the data in our possession, the assessment of UNESCO topics was made considering only if the topics had been addressed during the courses or not. Indeed, despite the high amount of information retrieved about the projects, it was not possible to evaluate the extent of the topics and the quality (in terms of “scientific validity”) of the sex education programs performed. The quality depends on several variables, such as the actual implementation of the program, the background of the actors who implement the program, or the political views of the association/institution that supports the program. However, it should be noted that all the programs included in the present analysis were implemented by local health authorities, who are affiliated with public institutions that should be neutral and avoid anti-scientific principles. Additionally, due to discrepancies between the number of projects carried out in northern, central, and southern Italy, the analyses may be biased. Therefore, it is not possible to say if these projects truly filled the gap in sex education between Italy and other European countries. Last, the rapid systematic review identified only three studies, limiting the inference about intervention efficacy. Nonetheless, this paper provides an updated overview of the main programs offered by institutions in Italy, thus showing the path of sex education in this country and suggesting potential topics and targets that should be further explored in Italian schools.

### Conclusions

In consideration of the picture that emerged from the analysis, it could be stated that in Italy, there is an important limitation regarding the dissemination of sex education. The presence of local initiatives funded by the local health authorities and the regions manages to only partially overcome these shortcomings. In addition, there is a large discrepancy between the northern and southern regions, with the latter being at a disadvantage.

In conclusion, the results of the present paper show that it is mandatory to increase the offer of sex education interventions, to reach adequate standards of education and to flatten the differences that exist not only between the various Italian regions but also between Italy and other European countries, in order to offer to the next generations adequate knowledge and competences about sex and sexuality and thus increase their well-being.

## Supplementary Information

Below is the link to the electronic supplementary material.

Supplementary file1 (DOCX 48 KB)

Supplementary file2 (DOCX 21 KB)

## Data Availability

All the material used for this research is available in Appendix B.

## References

[CR1] Benni, E., Sacco, S., Bianchi, L., Carrara, R., Zanini, C., Comelli, M., Tenconi, M. T., & SISM Educators Group (2016). Evaluation outcomes of a sex education strategy in high schools of Pavia (Italy). *Global Health Promotion*, *23*(2), 15–29. 10.1177/1757975914558309 10.1177/1757975914558309 25724750

[CR2] Bergamini M, Cucchi A, Guidi E, Stefanati A, Bonato B, Lupi S, Gregorio P (2013). Risk perception of sexually transmitted diseases and teenage sexual behavior: Attitudes towards in a sample of Italian adolescents. Journal of Preventive Medicine and Hygiene.

[CR3] Blanc Molina A, Rojas Tejada AJ (2018). Uso del preservativo, número de parejas y debut sexual en jóvenes en coito vaginal, sexo oral y sexo anal [Condom use, number of partners and sexual debut in young people in penile-vaginal intercourse, oral sex and anal sex]. Revista Internacional de Andrología.

[CR4] Bogani G, Cromi A, Serati M, Monti Z, Apolloni C, Nardelli F, Di Naro E, Ghezzi F (2015). Impact of school-based educational programs on sexual behaviors among adolescents in northern Italy. Journal of Sex & Marital Therapy.

[CR5] Borraccino, A., Moro, G. L., Dalmasso, P., Nardone, P., Donati, S., Berchialla, P., Charrier, L., Lenzi, M., Spinelli, A., Lemma, P., & the 2018 HBSC-Italia Group. (2020). Sexual behaviour in 15-year-old adolescents: Insights into the role of family, peer, teacher, and classmate support. *Annali dell'Istituto superiore di sanita*, *56*(4), 522–530. 10.4415/ANN_20_04_17 33346181

[CR6] European Commission. (2011). *Regions in the European Union-Nomenclature of territorial units for statistics-NUTS 2010/EU-27*. Available at: https://ec.europa.eu/eurostat/web/products-manuals-and-guidelines/-/ks-ra-11-011. Accessed Jan Dec 7, 2022.

[CR7] European Commission, Directorate-General for Employment, Social Affairs and Inclusion, Picken, N., (2021). *Sexuality education across the European Union: An overview*. Available at: https://data.europa.eu/doi/10.2767/869234.

[CR32] De Santi, A., Guerra, R., & Morosini, P. (Eds.). (2008). *La promozione della salute nelle scuole: obiettivi di insegnamento e competenze comuni*. Roma: Istituto Superiore di Sanità. (Rapporti ISTISAN 08/1). Available at: https://www.iss.it/documents/20126/45616/08-1_WEB.1204719565.pdf/8f0a0d06-30e2-022c-4224-db30743dca59?t=1581098517673 Accessed Feb 16, 2022.

[CR8] Del Prete G, Giraldi G, Miccoli S, Salamone V, Speranza M, Vita M, La Torre G (2012). Affettività e sessualità tra gli adolescenti: trial randomizzato sull'efficacia di un intervento di promozione della salute nella scuola secondaria di primo grado [Adolescents' affectivity and sexuality: a randomized trial of the efficacy of a school health promotion intervention in a primary school]. Igiene e Sanita Pubblica.

[CR9] Drago F, Ciccarese G, Zangrillo F, Gasparini G, Cogorno L, Riva S, Parodi A (2016). A survey of current knowledge on sexually transmitted diseases and sexual behaviour in Italian adolescents. International Journal of Environmental Research and Public Health.

[CR10] Epicentro. (n.d.). *Infezioni sessualmente trasmesse. Aspetti epidemiologici in Italia*. Available at: https://www.epicentro.iss.it/ist/epidemiologia-italia Accessed Oct 9, 2022

[CR11] Fontana, I. (2018). *Mapping sex and relationship education (SRE) in Italy*. Available at: https://gen-pol.org/2018/10/mapping-sex-and-relationship-education-sre-in-italy/ Accessed Oct 9, 2022.

[CR12] Future of Sex Education Initiative. (2012). *National sexuality education standards: Core content and skills, K-12*. Retrieved from http://www.futureofsexeducation.org/documents/josh-fose-standards-web.pdf. Accessed Feb 15, 2022.

[CR13] Garcia L, Fields J (2017). Renewed commitments in a time of vigilance: Sexuality education in the USA. Sex Education.

[CR14] García-Vázquez J, Quintó L, Agulló-Tomás E (2020). Impact of a sex education programme in terms of knowledge, attitudes and sexual behaviour among adolescents in Asturias (Spain). Global Health Promotion.

[CR15] Goldfarb ES, Lieberman LD (2020). Three decades of research: The case for comprehensive sex education. Journal of Adolescent Health.

[CR16] Helmer J, Senior K, Davison B, Vodic A (2015). Improving sexual health for young people: Making sexuality education a priority. Sex Education.

[CR17] Holland-Hall C, Quint EH (2017). Sexuality and disability in adolescents. Pediatric Clinics of North America.

[CR18] International Planned Parenthood Federation (IPPF), University of Lund and WHO Regional Office for Europe. (2006). *Sexuality education in Europe–a reference guide to policies and practices. The SAFE-Project*. London: IPPF. Available at: https://healtheducationresources.unesco.org/library/documents/sexuality-education-europe-reference-guide-policies-and-practices. Accessed Jan 11, 2022.

[CR19] Khangura S, Konnyu K, Cushman R, Grimshaw J, Moher D (2012). Evidence summaries: The evolution of a rapid review approach. Systematic Reviews.

[CR20] Kirby DB, Laris BA, Rolleri LA (2007). Sex and HIV education programs: Their impact on sexual behaviors of young people throughout the world. Journal of Adolescent Health.

[CR21] Lameiras-Fernández M, Martínez-Román R, Carrera-Fernández MV, Rodríguez-Castro Y (2021). Sex education in the spotlight: What is working? Systematic review. International Journal of Environmental Research and Public Health.

[CR22] Lindberg LD, Maddow-Zimet I, Boonstra H (2016). Changes in adolescents' receipt of sex education, 2006–2013. Journal of Adolescent Health.

[CR23] Magnusson BM, Crandall A, Evans K (2019). Early sexual debut and risky sex in young adults: The role of low self-control. BMC Public Health.

[CR24] OECD (2020). OECD regions and cities at a glance 2020.

[CR25] Orlando G, Campaniello M, Iatosti S, Grisdale PJ (2019). Impact of training conferences on high-school students’ knowledge of sexually transmitted infections (STIs). Journal of Preventive Medicine and Hygiene.

[CR26] Ouzzani M, Hammady H, Fedorowicz Z, Elmagarmid A (2016). Rayyan—a web and mobile app for systematic reviews. Systematic Reviews.

[CR27] Page MJ, McKenzie JE, Bossuyt PM, Boutron I, Hoffmann TC, Mulrow CD, Moher D (2021). The PRISMA 2020 statement: An updated guideline for reporting systematic reviews. BMJ.

[CR28] European Parliament, Directorate-General for Internal Policies of the Union, Beaumont, K., Maguire, M. (2013). *Policies for sexuality education in the European Union*. Available at: https://data.europa.eu/doi/10.2861/11317. Accessed Jan 11, 2022.

[CR29] Pastorelli, E., & Stocchiero, A. (2019). *Inequalities in Italy*. Available at: https://www.sdgwatcheurope.org/wp-content/uploads/2019/06/8.3.a-report-IT.pdf . Accessed Oct 9, 2022.

[CR30] Popay, J., Roberts, H., Sowden, A., Petticrew, M., Arai, L., Rodgers, M., Duffy, S. (2006). *Guidance on the conduct of narrative synthesis in systematic reviews. A product from the ESRC methods programme*. Version 1. ESRC.

[CR31] Pound P, Langford R, Campbell R (2016). What do young people think about their school-based sex and relationship education? A qualitative synthesis of young people’s views and experiences. British Medical Journal Open.

[CR33] Scaioli G, Bert F, Galis V, Brusaferro S, De Vito E, La Torre G, Siliquini R (2015). Pregnancy and internet: Sociodemographic and geographic differences in e-health practice. Results from an Italian multicenter study. Public Health.

[CR34] Smorti M, Milone A, Gonzalez Gonzalez J, Vitali Rosati G (2019). Adolescent selfie: An Italian Society of Paediatrics Survey of the Lifestyle of Teenagers. Italian Journal of Pediatrics.

[CR35] Stull, G. (2012). *Sexuality education in the EU-‘Sex education’ in a broader social context*. Available at https://www.europarl.europa.eu/thinktank/en/document/LDM_BRI(2012)120262. Accessed Jan 11, 2022.

[CR36] UNESCO. (2009). *International technical guidance on sexuality education: An evidence-informed approach for schools, teachers and health educators*. Available at: https://unesdoc.unesco.org/ark:/48223/pf0000183281. Accessed Jan 18, 2022.

[CR37] UNESCO. (2018). *Revised edition. International technical guidance on sexuality education: An evidence-informed approach*. Available at: https://unesdoc.unesco.org/ark:/48223/pf0000260770/. Accessed Jan 18, 2022.

[CR38] Vivancos R, Abubakar I, Phillips-Howard P, Hunter PR (2013). School-based sex education is associated with reduced risky sexual behavior and sexually transmitted infections in young adults. Public Health.

[CR39] World Health Organization (WHO). (2021). *Health promotion glossary of terms 2021*. Geneva. Available at: https://www.who.int/publications/i/item/9789240038349.

[CR40] World Health Organization (WHO). (n.d.). *Sexual and reproductive health and research*. Available at: https://www.who.int/teams/sexual-and-reproductive-health-and-research/key-areas-of-work/sexual-health/defining-sexual-health. Accessed Jan 10, 2022.

[CR41] Zizza A, Guido M, Recchia V, Grima P, Banchelli F, Tinelli A (2021). Knowledge, information needs and risk perception about HIV and sexually transmitted diseases after an education intervention on Italian high school and university students. International Journal of Environmental Research and Public Health.

