# Supplementary material for: Sex Education in Italy: An Overview of 15 Years of Projects in Primary and Secondary Schools
Source: Arch Sex Behav. 2023 Feb 7;52(4):1653–63. doi: 10.1007/s10508-023-02541-6 (PMC10125923; doi:10.1007/s10508-023-02541-6)
Supplement: Supplementary file 1 — Supplementary file1 (DOCX 48 KB) [file 10508_2023_2541_MOESM1_ESM.docx]

**Appendix A: Supplementary tables**

**Table A1.** Characteristics of each project (grey literature review)

| **POSITION IN ITALY** | **REGION** | **PROJECT NAME** | **YEAR/S OF IMPLEMENTATION** | **PROMOTING INSTITUTION** | **LOCATION** | **TARGET** | **FIGURES INVOLVED** | **TOTAL HOURS** |
| --- | --- | --- | --- | --- | --- | --- | --- | --- |
| South | Basilicata | Ben...Essere degli Studenti | 2007-2008; 2008-2009; 2009-2010 | ASL | counselling centre | lower secondary schools + upper secondary schools + parents + teachers | health professionals + psychologists + social assistants + sociologists | NA |
| South | Campania | "Ben...Essere In Amore" - Sessualità responsabile e prevenzione delle Malattie Sessualmente Trasmissibili | 2018-2019; 2019-2020 | ASL | school | lower secondary schools + upper secondary schools + parents + teachers | NA | 4 |
| North | Emilia-Romagna | W l’amore | 2013-2014 | Region | school | lower secondary schools | health professionals + psychologists | 20 |
| North | Friuli-Venezia Giulia | Affettività e sessualità in adolescenza: ilconsultorio familiare come riferimento per i ragazzi | 2016-2017 | ASL | counselling centre | upper secondary schools + teachers | health professionals | 8 |
| Centre | Lazio | Cantiere Scuola e Salute | 2006-2007 | ASL | school | lower secondary schools + upper secondary schools + parents + teachers | NA | NA |
| North | Lombardy | Sviluppo delle life skills in tema di affettività e sessualità | 2016-2017; 2017-2018; 2018-2019 | Region | school | parents + teachers | health professionals | 28 |
|  |  | Promozione del benessere dell'adolescente riguardo ad affettività e sessualità | 2017-2018; 2018-2019 | Region | school | upper secondary schools + teachers | health professionals | NA |
| South | Molise | Adolescenti e il sesso: quello che internet non vi spiega | 2017-2018; 2018-2019 | Region | school | upper secondary schools + teachers | health professionals | variable |
|  |  | "TTL: Talk to live" Non ballo da solo!!! Prevenzione delle Malattie Sessualmente Trasmesse (MST) | 2017-2018; 2018-2020 | Region | school | upper secondary schools | health professionals | variable |
| North | Piedmont | Educazione relazionale e affettiva Asti Sud | 2006-2007 | ASL | school | primary schools + lower secondary schools + upper secondary schools + teachers | NA | 3 |
|  |  | Per piacere, ci serve il sapere | 2008-2009 | ASL | school | primary schools + teachers | medical students + teachers | 7 |
|  |  | Educare alla sessualità: chi io? | 2008-2009 | ASL | school | teachers | health professionals + teachers | 6 |
|  |  | Laboratorio salute - progetto DMI - Consultorio - Sessualità Affettività | 2007-2008; 2008-2009 | ASL | school | primary schools + lower secondary schools + teachers | psychologists + teachers | 21 |
|  |  | "Io sono unico e speciale!": l'educazione alla sessualità come prevenzione dell'abuso | 2007-2008; 2008-2009; 2009-2010; 2010-2011 | ASL | school | primary schools + lower secondary schools + parents + teachers | health professionals + teachers | 182 |
|  |  | Educazione all’affettività autonomia e sessualità | 2010-2011; 2011-2012 | ASL | school | upper secondary schools | health professionals | 4 |
|  |  | Basta con api e fiorellini... (ma col cavolo che qualcuno glielo ha spiegato) | 2012-2013 | ASL | school | primary schools | NA | 4 |
|  |  | Educhiamo alla sessualità e all'affettività | 2012-2013; 2015-2016 | ASL | school | upper secondary schools | NA | 6 |
|  |  | Alla scoperta dell'...amore | 2014-2015; 2015-2016; 2019-2020 | ASL | school | upper secondary schools | NA | 4 |
|  |  | E se i bambini non nascessero sotto i cavoli? | 2006-2007; 2012-2013; 2019-2020 | ASL | school | lower secondary schools + upper secondary schools | health professionals | 8 |
|  |  | Ma che cavolo dici | 2011-2012; 2012-2013; 2013-2014; 2014-2015; 2015-2016; 2016-2017; 2017-2018; 2018-2019 | ASL | school | lower secondary schools | NA | 6 |
|  |  | Educazione alla sessualità: con-tatto con affetto | 2016-2017; 2017-2018; 2018-2019; 2019-2020 | ASL | school | primary schools + teachers | NA | 6 |
|  |  | I passi dell'amore - progetto pilota | 2019-2020 | ASL | school | primary schools | health professionals | 3 |
|  |  | Cavoli e Cicogne. Percorso di educazione sessuale nella scuola primaria secondo il metodo narrativo | 2006-2007; 2007-20008; 2008-2009; 2009-2010; 2010-2011; 2011-2012; 2012-2013; 2013-2014; 2014-2015; 2015-2016; 2016-2017; 2017-2018; 2018-2019; 2019-2020 | ASL | school | primary schools + lower secondary schools + upper secondary schools + teachers | NA | 12 |
|  |  | L'amore è | 2018-2019; 2019-2020 | ASL | school | primary schools + lower secondary schools | health professionals | 48 |
| North | Trentino-South Tyrol | Progetto di educazione socio-affettiva e sessuale | 2008-2009 | ASL | school | lower secondary schools + upper secondary schools | health professionals + psychologists | 6 |
| Centre | Umbria | Educazione all’affettività e sessualità nelle scuole | 2006-2007 | ASL | school | lower secondary schools + upper secondary schools + parents + teachers | NA | 3 |
|  |  | Il tempo del cerchio | 2007-2008 | ASL | school | lower secondary schools + upper secondary schools + parents + teachers | health professionals | 10 |
| North | Aosta Valley | Impara l'abc | 2012-2013 | ASL | school | lower secondary schools + upper secondary schools + parents + teachers | Italian Red Cross | 8 |
|  |  | Edusex - psicologia sessuale 1 | 2012-2013 | ASL | school | primary schools | psychologists | 12 |
|  |  | Cambiamenti - psicologia sessuale 2 | 2012-2013 | ASL | school | lower secondary schools | psychologists | 2 |
|  |  | Sessual...Mentis | 2012-2013 | ASL | school | lower secondary schools + upper secondary schools + parents + teachers | health professionals + psychologists | 12 |
|  |  | Educazione alla sessualità | 2012-2013 | ASL | school | upper secondary schools | NA | NA |
|  |  | Promozione del benessere dell'adolescente riguardo ad affettività e sessualità del disabile | 2012-2013 | ASL | counselling centre | lower secondary schools + upper secondary schools | health professionals | variable |
|  |  | Promozione del benessere dell'adolescente riguardo ad affettività e sessualità | 2012-2013 | ASL | school | upper secondary schools + teachers | health professionals | variable |
| North | Veneto | Siamo noi la meglio gioventù. Curiosi, confusi, a confronto sul sesso e sull’amore | 2017-2018 | Region | school | lower secondary schools + upper secondary schools + parents + teachers | health professionals + psychologists + social assistants | 12 |
|  |  | Gentili atti d’amore + La sessualità è uguale per tutti | 2017-2018 | Region | school | upper secondary schools | health professionals + psychologists + social assistants | 12 |
|  |  | La mediazione sessuale | 2017-2018 | Region | school | teachers | health professionals + psychologists + social assistants | 9 |
|  |  | Disabilità fa rima con sessualità? | 2017-2018 | Region | school | teachers | health professionals + psychologists + social assistants | 12 |
|  |  | ...E me lo chiami amore? | 2017-2018 | Region | school | parents | health professionals + psychologists + social assistants | NA |

Abbreviations: ASL=Azienda Sanitaria Locale; NA=Not Available.

**Table A2.** Topics in each project (grey literature review)

| **PROJECT NAME** | **BIOLOGICAL ASPECTS/BODY AWARENESS/PUBERTY AND ANATOMY** | **LOVE, MARRIAGE, PARTNERSHIPS, FAMILY** | **SEXUAL/DOMESTIC ABUSE AND GENDER-BASED VIOLENCE** | **PREGNANCY AND BIRTH** | **SEXUAL ORIENTATION / LGBTI ISSUES** | **HIV/AIDS AND STIS** | **CONTRACEPTION** | **GENDER ROLES** | **MUTUAL CONSENT** | **HUMAN RIGHTS** | **ONLINE MEDIA** | **DISABILITY** | **FINAL EVALUATION** |
| --- | --- | --- | --- | --- | --- | --- | --- | --- | --- | --- | --- | --- | --- |
| Ben...Essere degli Studenti | **X** | **X** | **X** | **X** |  | **X** | **X** |  | **X** | **X** |  |  |  |
| "Ben...Essere In Amore" - Sessualità responsabile e prevenzione delle Malattie Sessualmente Trasmissibili | **X** | **X** | **X** |  | **X** | **X** | **X** | **X** | **X** | **X** | **X** |  | **X** |
| W l’amore | **X** | **X** |  |  |  | **X** | **X** | **X** |  |  |  |  | **X** |
| Affettività e sessualità in adolescenza: il consultorio familiare come riferimento per i ragazzi | **X** | **X** |  |  |  | **X** | **X** |  |  |  |  |  | **X** |
| Cantiere Scuola e Salute |  |  |  |  |  | **X** |  |  |  |  |  |  |  |
| Sviluppo delle life skills in tema di affettività e sessualità |  | **X** |  |  |  |  | **X** |  |  |  |  |  | **X** |
| Promozione del benessere dell'adolescente riguardo ad affettività e sessualità |  | **X** |  |  |  | **X** | **X** |  |  |  |  |  | **X** |
| Adolescenti e il sesso: quello che internet non vi spiega | **X** |  |  |  |  | **X** | **X** |  |  |  |  |  |  |
| "TTL: Talk to live" Non ballo da solo!!! Prevenzione delle Malattie Sessualmente Trasmesse (MST) |  | **X** |  |  |  | **X** | **X** |  |  |  | **X** |  | **X** |
| Educazione relazionale e affettiva Asti Sud | **X** | **X** |  |  | **X** |  |  |  |  |  |  |  | **X** |
| Per piacere, ci serve il sapere |  |  |  |  |  |  | **X** | **X** |  |  |  |  | **X** |
| Educare alla sessualità: chi io? | **X** | **X** |  |  |  |  |  |  |  |  |  |  | **X** |
| Laboratorio salute - progetto DMI - Consultorio - Sessualità Affettività | **X** |  |  | **X** |  |  | **X** |  |  |  |  |  |  |
| "Io sono unico e speciale!": l'educazione alla sessualità come prevenzione dell'abuso |  | **X** | **X** |  |  |  |  |  |  |  |  |  |  |
| Educazione all’affettività autonomia e sessualità |  | **X** |  |  |  | **X** | **X** |  |  |  |  |  |  |
| Basta con api e fiorellini... (ma col cavolo che qualcuno glielo ha spiegato) | **X** | **X** | **X** |  |  |  |  |  |  |  |  |  | **X** |
| Educhiamo alla sessualità e all'affettività | **X** | **X** | **X** |  | **X** |  | **X** |  |  |  |  |  |  |
| Alla scoperta dell'...amore | **X** | **X** |  | **X** | **X** | **X** | **X** | **X** |  |  |  |  |  |
| E se i bambini non nascessero sotto i cavoli? | **X** | **X** |  | **X** |  | **X** | **X** |  |  |  |  |  |  |
| Ma che cavolo dici | **X** | **X** |  | **X** |  |  |  |  |  |  |  |  | **X** |
| Educazione alla sessualità: con-tatto con affetto | **X** | **X** |  | **X** |  | **X** | **X** | **X** |  |  |  |  | **X** |
| I passi dell'amore - progetto pilota |  | **X** |  |  | **X** | **X** |  | **X** |  |  |  |  | **X** |
| Cavoli e Cicogne. Percorso di educazione sessuale nella scuola primaria secondo il metodo narrativo |  | **X** |  |  | **X** |  |  | **X** |  |  |  |  | **X** |
| L'amore è | **X** | **X** |  |  | **X** | **X** | **X** |  |  |  |  |  | **X** |
| Progetto di educazione socio-affettiva e sessuale | **X** | **X** |  |  |  | **X** | **X** |  |  | **X** |  |  | **X** |
| Educazione all’affettività e sessualità nelle scuole | **X** | **X** |  |  |  |  | **X** |  | **X** |  |  |  | **X** |
| Il tempo del cerchio | **X** |  |  |  | **X** |  |  | **X** |  |  |  |  | **X** |
| Impara l'abc |  |  |  | **X** |  | **X** | **X** |  |  |  |  |  | **X** |
| Edusex - psicologia sessuale 1 | **X** |  |  | **X** |  |  |  |  |  |  | **X** |  | **X** |
| Cambiamenti - psicologia sessuale 2 | **X** |  |  | **X** |  |  |  |  |  |  |  |  | **X** |
| Sessual...Mentis | **X** | **X** | **X** | **X** | **X** | **X** |  | **X** | **X** | **X** |  |  | **X** |
| Educazione alla sessualità | **X** |  | **X** | **X** | **X** | **X** |  | **X** |  |  |  |  | **X** |
| Promozione del benessere dell'adolescente riguardo ad affettività e sessualità del disabile | **X** | **X** | **X** |  | **X** |  | **X** | **X** |  |  |  | **X** | **X** |
| Promozione del benessere dell'adolescente riguardo ad affettività e sessualità |  | **X** |  |  |  | **X** | **X** |  |  |  |  |  | **X** |
| Siamo noi la meglio gioventù. Curiosi, confusi, a confronto sul sesso e sull’amore | **X** | **X** | **X** |  | **X** |  | **X** | **X** |  |  |  |  |  |
| Gentili atti d’amore + La sessualità è uguale per tutti | **X** | **X** | **X** |  | **X** |  | **X** | **X** |  |  |  | **X** |  |
| La mediazione sessuale | **X** | **X** | **X** |  |  |  |  |  |  |  | **X** |  |  |
| Disabilità fa rima con sessualità? |  |  |  |  |  |  |  |  |  |  |  | **X** |  |
| ...E me lo chiami amore? | **X** |  |  |  |  |  |  |  |  |  |  |  |  |

**Table A3.** Characteristics of selected papers (rapid systematic review)

| **TITLE** | **YEAR OF PUBLICATION** | **POSITION** | **YEARS** | **LOCATION** | **TARGET** | **FIGURES INVOLVED** | **TOTAL HOURS** | **SAMPLE SIZE** | **INTERVENTION** |
| --- | --- | --- | --- | --- | --- | --- | --- | --- | --- |
| Adolescents' affectivity and sexuality: a randomized trial of the efficacy of a school health promotion intervention in a primary school | 2012 | South | 2011-2012 | school | lower secondary schools | health professionals | 5 | Experimental group: 147  Control group: 175 | classes + survey |
| Evaluation outcomes of a sex education strategy in upper secondary schools of Pavia (Italy) | 2016 | North | 2011-2012 | school | upper secondary schools | medical students + postgraduate students + academic researchers | 4 | Experimental group: 547  Control group: 355 | classes + survey |
| Knowledge, Information Needs and Risk Perception about HIV and Sexually Transmitted Diseases after an Education Intervention on Italian Upper secondary school and University Students | 2021 | South | 2018-2019 | school | upper secondary schools | experts | NA | Experimental group: 230  Control group: none | classes + survey |

Abbreviations: NA=Not Available

**Table A4.** Topics in each selected paper (rapid systematic review)

| **TITLE** | **BIOLOGICAL ASPECTS/BODY AWARENESS/PUBERTY AND ANATOMY** | **LOVE, MARRIAGE, PARTNERSHIPS, FAMILY** | **SEXUAL/DOMESTIC ABUSE AND GENDER-BASED VIOLENCE** | **PREGNANCY AND BIRTH** | **SEXUAL ORIENTATION / LGBTI ISSUES** | **HIV/AIDS AND STIS** | **CONTRACEPTION** | **GENDER ROLES** | **MUTUAL CONSENT** | **HUMAN RIGHTS** | **ONLINE MEDIA** | **DISABILITY** |
| --- | --- | --- | --- | --- | --- | --- | --- | --- | --- | --- | --- | --- |
| Adolescents' affectivity and sexuality: a randomized trial of the efficacy of a school health promotion intervention in a primary school | **X** | **X** | **X** |  |  | **X** | **X** |  | **X** |  | **X** |  |
| Evaluation outcomes of a sex education strategy in high schools of Pavia (Italy) | **X** |  |  |  |  | **X** | **X** |  |  |  |  |  |
| Knowledge, Information Needs and Risk Perception about HIV and Sexually Transmitted Diseases after an Education Intervention on Italian High School and University Students |  |  |  |  |  | **X** |  |  |  |  |  |  |
