# Supplementary material for: Sex Education in Italy: An Overview of 15 Years of Projects in Primary and Secondary Schools
Source: Arch Sex Behav. 2023 Feb 7;52(4):1653–63. doi: 10.1007/s10508-023-02541-6 (PMC10125923; doi:10.1007/s10508-023-02541-6)
Supplement: Supplementary file 2 — Supplementary file2 (DOCX 21 KB) [file 10508_2023_2541_MOESM2_ESM.docx]

**Appendix B: references of all projects**

**Table B1.** References of each project (grey literature review)

| **REGION** | **PROJECT NAME** | **PROMOTING INSTITUTION** | **LAST UPDATE** | | **ACCESSED** | **AVAILABLE AT** |
| --- | --- | --- | --- | --- | --- | --- |
| Basilicata | Ben...Essere degli Studenti | ASL | | NA | Dec 2021 | http://www.aspbasilicata.it/sites/default/files/PROGETTO%20BEN%20ESSERE.pdf |
| Campania | "Ben...Essere In Amore" - Sessualità responsabile e prevenzione delle Malattie Sessualmente Trasmissibili | ASL | | NA | Dec 2021 | https://www.aslsalerno.it/moduli/output_immagine.php?id=5349 |
| Emilia-Romagna | W l’amore | Region | | 28/10/2015 | Dec 2021 | retepromozionesalute.it/bd2_scheda.php?idpr2=3392 |
| Friuli-Venezia Giulia | Affettività e sessualità in adolescenza: il consultorio familiare come riferimento per i ragazzi | ASL | | NA | Dec 2021 | http://www.fondazionecarigo.it/proxyvfs.axd/null/r24514/catalogo-benessere-progetti-ed-interventi-di-promozione-e-di-educazione-alla-salute-nella-scuola-pdf?ext=.pdf&v=15824 |
| Lazio | Cantiere Scuola e Salute | ASL | | 2006 | Dec 2021 | https://www.retepromozionesalute.it/scuola_scheda.php?idpr2=492 |
| Lombardy | Sviluppo delle life skills in tema di affettività e sessualità | Region | | 04/03/2020 | Dec 2021 | retepromozionesalute.it/bd2_scheda.php?idpr2=4619 |
|  | Promozione del benessere dell'adolescente riguardo ad affettività e sessualità | Region | | 06/02/2019 | Dec 2021 | https://www.retepromozionesalute.it/bd2_scheda.php?idpr2=4499 |
| Molise | Adolescenti e il sesso: quello che internet non vi spiega | Region | | NA | Dec 2021 | http://www3.regione.molise.it/flex/cm/pages/ServeAttachment.php/L/IT/D/8%252Fc%252Fd%252FD.d2be508786ce780f1570/P/BLOB%3AID%3D129/E/pdf |
|  | "TTL: Talk to live" Non ballo da solo!!! Prevenzione delle Malattie Sessualmente Trasmesse (MST) | Region | | 02/05/2018 | Dec 2021 | http://www3.regione.molise.it/flex/cm/pages/ServeAttachment.php/L/IT/D/8%252Fc%252Fd%252FD.d2be508786ce780f1570/P/BLOB%3AID%3D129/E/pdf |
| Piedmont | Educazione relazionale e affettiva Asti Sud | ASL | | 07/07/2010 | Dec 2021 | https://www.retepromozionesalute.it/bd2_scheda_arc.php?idpr2=252 |
|  | Per piacere, ci serve il sapere | ASL | | 16/05/2012 | Dec 2021 | https://www.retepromozionesalute.it/bd2_scheda_arc.php?idpr2=325 |
|  | Educare alla sessualità: chi io? | ASL | | 29/07/2010 | Dec 2021 | https://www.retepromozionesalute.it/bd2_scheda_arc.php?idpr2=2640 |
|  | Laboratorio salute - progetto DMI - Consultorio - Sessualità Affettività | ASL | | 04/06/2012 | Dec 2021 | https://www.retepromozionesalute.it/bd2_scheda_arc.php?idpr2=2898 |
|  | "Io sono unico e speciale!": l'educazione alla sessualità come prevenzione dell'abuso | ASL | | 15/03/2013 | Dec 2021 | https://www.retepromozionesalute.it/bd2_scheda_arc.php?idpr2=263 |
|  | Educazione all’affettività autonomia e sessualità | ASL | | 03/02/2016 | Dec 2021 | https://www.retepromozionesalute.it/bd2_scheda_arc.php?idpr2=3166 |
|  | Basta con api e fiorellini... (ma col cavolo che qualcuno glielo ha spiegato) | ASL | | 03/06/2013 | Dec 2021 | https://www.retepromozionesalute.it/bd2_scheda_arc.php?idpr2=2976 |
|  | Educhiamo alla sessualità e all'affettività | ASL | | 01/03/2016 | Dec 2021 | https://www.retepromozionesalute.it/bd2_scheda.php?idpr2=2879 |
|  | Alla scoperta dell'...amore | ASL | | 02/09/2016 | Dec 2021 | https://www.retepromozionesalute.it/bd2_scheda.php?idpr2=2495 |
|  | E se i bambini non nascessero sotto i cavoli? | ASL | | 05/08/2014 | Dec 2021 | https://www.retepromozionesalute.it/bd2_scheda.php?idpr2=350 |
|  | Ma che cavolo dici | ASL | | 13/12/2019 | Dec 2021 | https://www.retepromozionesalute.it/bd2_scheda.php?idpr2=2788 |
|  | Educazione alla sessualità: con-tatto con affetto | ASL | | 04/04/2022 | Dec 2021 | https://www.retepromozionesalute.it/bd2_scheda.php?idpr2=3287 |
|  | I passi dell'amore - progetto pilota | ASL | | 13/01/2021 | Dec 2021 | https://www.retepromozionesalute.it/bd2_scheda.php?idpr2=5811 |
|  | Cavoli e Cicogne. Percorso di educazione sessuale nella scuola primaria secondo il metodo narrativo | ASL | | 04/03/2020 | Dec 2021 | https://www.retepromozionesalute.it/bd2_scheda.php?idpr2=1787 |
|  | L'amore è | ASL | | 31/01/2022 | Dec 2021 | https://www.retepromozionesalute.it/bd2_scheda.php?idpr2=3644 |
| Trentino-South Tyrol | Progetto di educazione socio-affettiva e sessuale | ASL | | NA | Dec 2021 | https://www.apss.tn.it/Azienda/Operatori-e-partner/Progetti-nelle-scuole/Scuola-secondaria-di-primo-grado/Edu-chi-amo.-Educazione-relazionale-affettiva-e-sessuale |
| Umbria | Educazione all’affettività e sessualità nelle scuole | ASL | | 01/10/2008 | Dec 2021 | https://www.retepromozionesalute.it/bd2_scheda_arc.php?idpr2=1529 |
|  | Il tempo del cerchio | ASL | | 15/02/2009 | Dec 2021 | https://www.retepromozionesalute.it/bd2_scheda_arc.php?idpr2=1555 |
| Aosta Valley | Impara l'abc | ASL | | NA | Dec 2021 | http://www.regione.vda.it/gestione/gestione_contenuti/allegato.asp?pk_allegato=12307 |
|  | Edusex - psicologia sessuale 1 | ASL | | NA | Dec 2021 | http://www.regione.vda.it/allegato.aspx?pk=25535 |
|  | Cambiamenti - psicologia sessuale 2 | ASL | | NA | Dec 2021 | https://www.regione.vda.it/gestione/gestione_contenuti/allegato.asp?pk_allegato=12299 |
|  | Sessual...Mentis | ASL | | NA | Dec 2021 | http://www.regione.vda.it/allegato.aspx?pk=25538 |
|  | Educazione alla sessualità | ASL | | 25/07/2011 | Dec 2021 | http://www.regione.vda.it/gestione/gestione_contenuti/allegato.asp?pk_allegato=12320 |
|  | Promozione del benessere dell'adolescente riguardo ad affettività e sessualità del disabile | ASL | | NA | Dec 2021 | https://www.regione.vda.it/gestione/gestione_contenuti/allegato.asp?pk_allegato=12319 |
|  | Promozione del benessere dell'adolescente riguardo ad affettività e sessualità | ASL | | NA | Dec 2021 | https://www.regione.vda.it/gestione/gestione_contenuti/allegato.asp?pk_allegato=12318 |
| Veneto | Siamo noi la meglio gioventù. Curiosi, confusi, a confronto sul sesso e sull’amore | Region | | 12/09/2017 | Dec 2021 | https://www.bestatreviso.edu.it/attachments/article/2352/Superiori%20progetti%2017-18%20(1).pdf |
|  | Gentili atti d’amore + La sessualità è uguale per tutti | Region | | 12/09/2017 | Dec 2021 | https://www.bestatreviso.edu.it/attachments/article/2352/Superiori%20progetti%2017-18%20(1).pdf |
|  | La mediazione sessuale | Region | | 12/09/2017 | Dec 2021 | https://www.bestatreviso.edu.it/attachments/article/2352/Superiori%20progetti%2017-18%20(1).pdf |
|  | Disabilità fa rima con sessualità? | Region | | 12/09/2017 | Dec 2021 | https://www.bestatreviso.edu.it/attachments/article/2352/Superiori%20progetti%2017-18%20(1).pdf |
|  | ...E me lo chiami amore? | Region | | 12/09/2017 | Dec 2021 | https://www.bestatreviso.edu.it/attachments/article/2352/Superiori%20progetti%2017-18%20(1).pdf |

Abbreviations: NA=Not Available
